# An Analysis of Genetic Changes during the Divergence of *Drosophila* Species

**DOI:** 10.1371/journal.pone.0010485

**Published:** 2010-05-05

**Authors:** Rui Sousa-Neves, Alexandre Rosas

**Affiliations:** 1 Department of Biology, Case Western Reserve University, Cleveland, Ohio, United States of America; 2 Departamento de Física, CCEN, Universidade Federal da Paraíba, João Pessoa, Paraíba, Brazil; Institute of Infectious Disease and Molecular Medicine, South Africa

## Abstract

**Background:**

It has been long appreciated that speciation involves changes in body plans and establishes genetic, reproductive, developmental and behavioral incompatibilities between populations. However, little is still known about the genetic components involved in these changes or the sequence and scale of events that lead to the differentiation of species.

**Principal Findings:**

In this paper, we investigated the genetic changes in three closely related species of *Drosophila* by making pair-wise comparisons of their genomes. We focused our analysis on the modern relatives of the alleles likely to be segregating in pre-historic populations at the time or after the ancestor of *D. simulans* became separated from the ancestor of *D. melanogaster*. Some of these genes were previously implicated in the genetics of reproduction and behavior while the biological functions of others are not yet clear.

**Conclusions:**

Together these results identify different classes of genes that might have participated in the beginning of segregation of these species millions of years ago in Africa.

## Introduction

One of the greatest challenges in modern Biology is the identification of genes that operate during evolution and diversification of species. However, since species are already separated and preserved samples of the prehistoric species are scarce, the investigation of the scale and types of genetic changes occurred during and after speciation has been limited.

The problem of speciation has puzzled generations of biologists and is of great significance to a wide variety of fields in biology. For instance, during evolution, mutations modify the architecture of brains and external appearances, novel behaviors appear and reciprocal lethal/sterile genetic systems emerge to block gene flow. The recurrent appearance of these themes across different phyla suggests a conservation of genetic processes. However, little is still known about the scale of the genetic changes that occur during speciation.

To investigate this issue, we chose to use the genetic workhorse *Drosophila melanogaster* and two sequenced sibling species, *D. simulans* and *D. sechellia*. The fact that *D. melanogaster* is a close relative of *D. simulans* and *D. sechellia* and has a myriad of genetic and genomic resources, makes it an ideal model to study evolutionary processes. The latest estimates suggest that *D. simulans* diverged from *D. melanogaster* approximately 5.4 million years ago in Africa, while *D. sechellia* diverged from *D. simulans* 0.5 million years ago in the Indian island of Seychelles [1]. Similar estimates suggest that a fourth species, the incompletely sequenced *D. mauritiana*, diverged from *D. simulans* 0.1–0.3 million years ago in the island of Mauritius.

Anatomically, these sibling species are almost identical, except for the different appearance of the male genitalia in all four species and minor ambiguous morphological features [2]. All four species have a set of 4 chromosomes mostly homosequential, but *D. simulans*, *D. sechellia* and *D. mauritiana* have nearly identical rearrangements that distinguish them from *D. melanogaster* [3]–[6]. These rearrangements suggest that the common ancestor of these species had already diverged chromosomally from *D. melanogaster* between 0.5 and 5.4 Million years ago [3]–[6].

Despite their similarities, the three sibling species (*D. melanogaster*, *D. simulans* and *D. sechellia*) have an intriguing different biology. For instance, the mating between *D. simulans* males and *D. melanogaster* females results in a dramatic larval death of the male offspring that is accompanied by a reduction of the brain and lack of imaginal discs [7], [8], while the surviving adult females are sterile [3], [9]. The reciprocal mating between *D. melanogaster* males and *D. simulans* or *D. sechellia* females is rarely successful and results in embryonic lethality of female and sterility of male offspring [3], [10], [11]. Similar results are obtained with hybrids between *D. sechellia* and *D. melanogaster* and somewhat less extreme phenotypes with *D. sechellia* and *D. simulans* hybrids. In the latter case, both sexes survive, but the male progeny is sterile [3]. Behaviorally, these species exhibit a mating asymmetry and it has been proposed that females of the newest species (i.e. *D. simulans* and *D. sechellia*) reject males of the oldest species archetype (i.e. *D. melanogaster*). In contrast, females of the oldest species accept males of the newest species [3], [12], [13]. Similar mating asymmetries appear in a significant number of closely related species [14].

Together, the facts highlighted above suggest that *D. simulans* and *D. sechellia* are more closely related to each other than to *D. melanogaster*, and quite conceivably share incompatible genes that affect mitotic, embryonic, reproductive, sensory perception and behavioral systems. However, with few notable exceptions, the genes and alleles involved in these processes still remain elusive [15]–[19].

Here we screened the genome for genetic variants that might reveal the changes occurred during or after the divergence of the ancestor of *D. simulans* from the ancestor of *D. melanogaster*. For convenience, *D. melanogaster* is taken as the archetypical or ancestral form as previously suggested [5], [12]. In particular, we searched for alleles with little or no divergence between *D. simulans* and *D. sechellia* that greatly diverged from *D. melanogaster*. These alleles are expected to have appeared at the time or right after the divergence of the ancestor of *D. simulans* from the ancestor of *D. melanogaster*, but before the separation of *D. simulans* from *D. sechellia*. For this reason, we refer to them as *ancestral alleles*. It is noteworthy that ancestral alleles of *D. simulans* and *D. sechellia*, also happen to be fast evolving alleles, when the Melanogaster subgroup is used as a reference. The analysis of the predicted gene products of ancestral alleles reveal which classes of genes might have been involved in the segregation of these species.

## Results

### Number of coding sequences identified in *D. simulans* and *D. sechellia*

The major objective of this search was to quantify and identify alleles that might have been segregating in pre-historic populations of the ancestor of *D. simulans* that were inherited by the descendants *D. simulans* and *D. sechellia*. We expected that ancestral alleles should be informative of the developmental, reproductive and behavioral novelties that distinguish *D. simulans* and *D. sechellia* from *D. melanogaster*.

**Figure 1:**
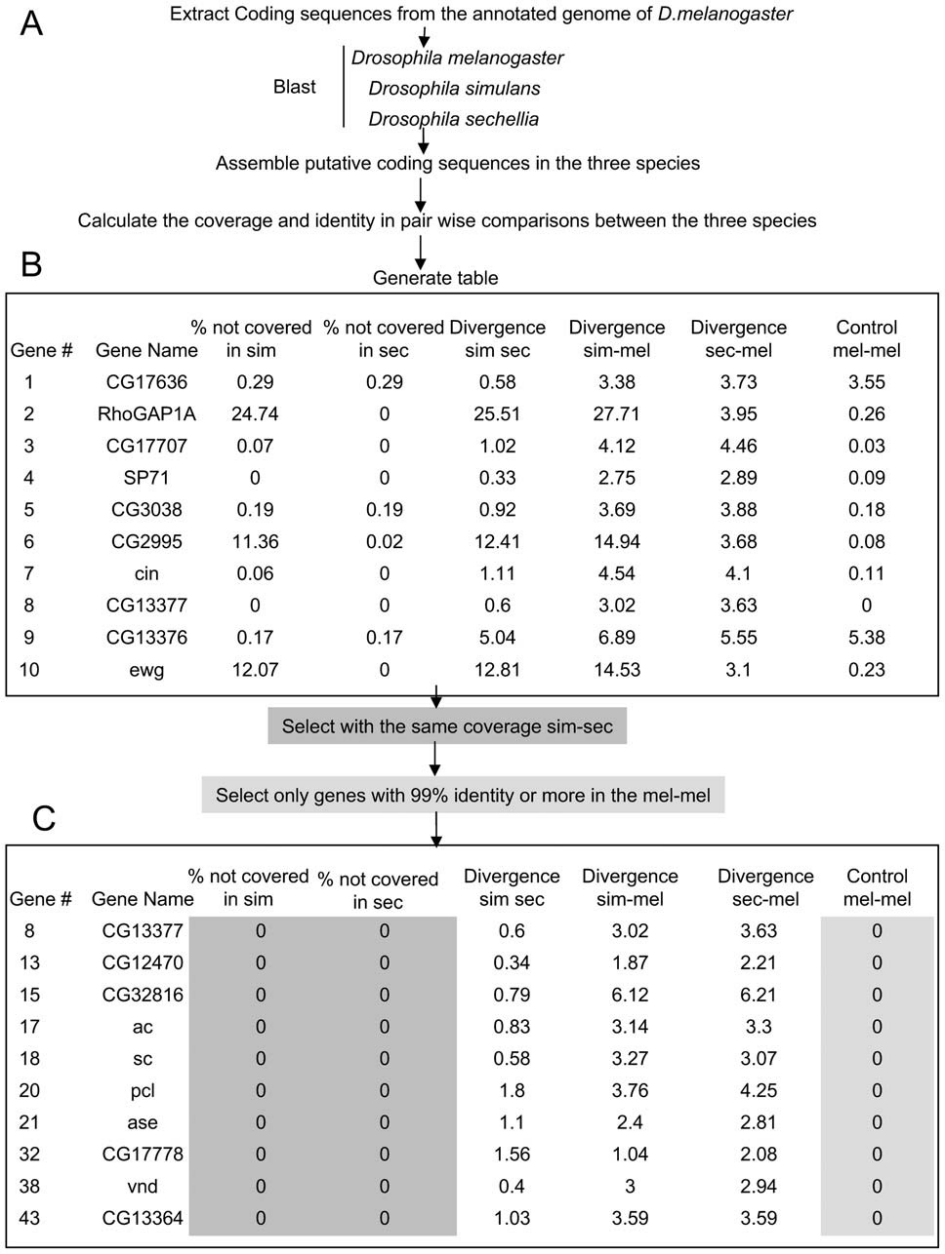
Overview of the data collection and sorting. A) Exons of coding sequence were extracted from the annotated genome of *D. melanogaster* using Extractor and electronically joined using Analyst to obtain complete coding sequences. These coding sequences were then automatically blasted against the genome of *D. melanogaster*, *D. simulans* and *D. sechellia* with Megablast. Analyst scanned the resulting alignments for the best hits and assembled the coding sequences in the three species from them. B) Analyst also calculated the coverage, the percentage not covered, the divergence in sim-sec, sim-mel, sec-mel as well as the control mel-mel and organized this data in a table. C) To minimize artifacts due to incomplete clone representation in the genomic libraries, the coding sequences were filtered and only genes with the same coverage in *D. simulans* and *D. sechellia* retrieved. To avoid genes truncated by Megablast (i.e. usually genes with small exons), only genes with a mismatch up to 1% in the control mel-mel were retrieved. After these two filters were applied, a new table like the one exemplified in C) was generated for each chromosome.

To begin addressing this issue, we extracted and compared the annotated coding sequences of *D. melanogaster* to the sequence of computationally predicted coding sequences of *D. simulans* and *D. sechellia* (Fig. 1A). A total of 13,740 predicted coding sequences were assembled from *D. simulans* and *D. sechellia* genomes: 2,226 on the X chromosome, 5,355 on the second chromosome, 6,074 on the third chromosome and 85 on the fourth chromosome.

### Identification of high confidence genes by sorting and filtering data

The data collected from each chromosome arm was organized in a table, which consists of eight columns with the following information: (1) coding sequence number in *D. melanogaster*; (2) gene name; (3) percentage of bases not covered in *D. simulans* and (4) in *D. sechellia*; (5) divergence between *D. simulans* and *D. sechellia*; (6) divergence between *D. melanogaster* and *D. simulans*; (7) divergence between *D. melanogaster* and *D. sechellia*; and finally, (8) assembly control (i.e. percentage of mismatches between the actual coding sequences of *D. melanogaster* vs. the coding sequences assembled from Blast results in *D. melanogaster*).

To avoid false positives due to truncated fragments in the WGS libraries, we first applied a filter that discards all genes with different coverage in *D. simulans* and *D. sechellia* (Fig. 1C, Columns 3 and 4). The remaining genes were sorted using values of the control Blast mel vs. mel (Fig. 1C, Column 8) in ascending order. In addition, only genes with a mismatch of up to 1% were retrieved. Our control of automatic assembly of coding sequences assured that only high quality coding sequences assembled from Blast alignments (i.e. 99% match or greater) were analyzed. After filtering the data using the criteria above, 8,416 reliable coding sequences corresponding to 61% of the total number of coding sequences extracted from *D. melanogaster* were obtained: 1,039 on the X; 3,407 on the second; 3,951 on the third and 19 on the fourth chromosome.

### Identification of genes that vary the least between *D. simulans* and *D. sechellia* and the most in *D. melanogaster*

To identify genes that diverged the least in the pair sim-sec and the most in the pairs sim-mel and sec-mel (ancestral alleles), we employed two strategies. The first strategy selects genes in the pair sim-sec that diverged less than the average plus the standard deviation of all genes in the same chromosome, and in addition that also diverged more than the average plus the standard deviation in sim-mel and sec-mel pairs. The second method, which will be explained in more detail in the next section, is based on the observation that most of the genes in *D. simulans*/*D. sechellia* diverge linearly from *D. melanogaster*, while few very similar genes diverge non-linearly.

**Figure 2:**
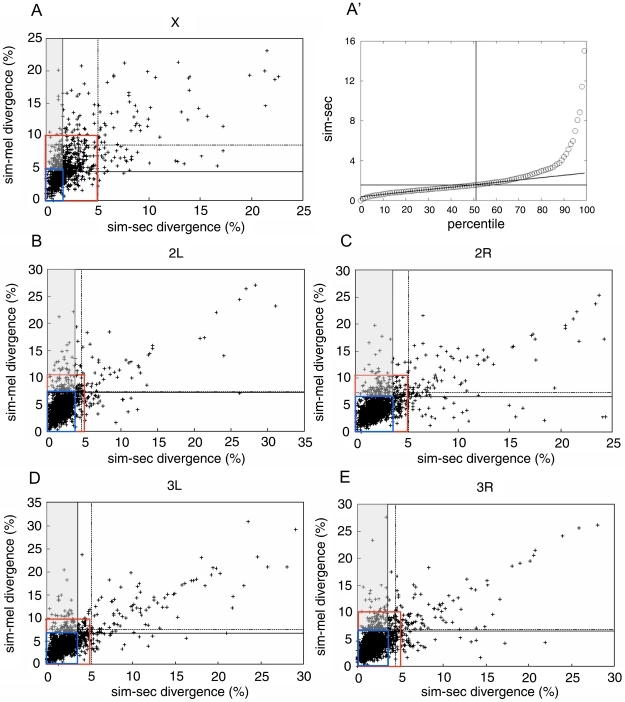
Patterns of divergence along chromosomes and two screening methods. A, B, C, D and E) Each graph corresponds to a chromosome or chromosome arm (X, 2L, 2R, 3L and 3R), where the genes are ordered from the least divergent to the most divergent in sim-sec (abscissa) and sim-mel (ordinate). The horizontal and vertical dashed lines delimit the averages plus one standard deviation of the divergence between sim-sec (horizontal) and sim-mel (vertical). The upper left quadrants delimit genes found by the method of averages and standard deviations. Note that the divergences of most genes in all 5 graphs are clustered in a quadrant that can be roughly delimited between the abscissa values of 0%–5% and ordinate values 0%–10% (red rectangles). In this quadrant, the genes have a good fit with a linear distribution (P<0.0001). To better delimit the quadrant in which the divergence is linear in each chromosome, the data was divided in percentiles of divergences of sim-sec, sim-mel and sec-mel. (A') exemplifies the percentiles of the X chromosome. Since each point in these curves represents one percentile, the percentage of genes that diverge linearly is equal to the number of points that can be transected by a straight line. Once this linear interval is defined, the values on the x and y axes become known and can be used to redefine the quadrant of linear divergences (inferior left quadrant in blue). The region where the genes in sim-sec vary the least and the genes in sim-mel vary the most is the adjacent upper quadrant to the left of the point where the horizontal and vertical lines cross (gray quadrant).

Out of the total 8,416 reliable coding sequences selected previously, the first method led to the identification of 517 genes: 67 genes on the X; 112 on the left arm of the second; 106 on the right arm of the second; 88 on the left arm of the third; and 144 on the right arm of the third chromosome (Table 1, Fig. 2). No ancestral alleles were identified on the fourth chromosome due to the fact this chromosome has highly homogeneous divergences (data not shown).

**Table 1:** Number of genes identified by the screening methods 1 and 2.

	X	2L	2R	3L	3R	Totals
Screening 1	67	112	106	88	144	517
Screening 2	101	74	89	77	98	439
Common	14	73	76	61	96	320

The genes identified by method 1 have divergences inferior to the average plus standard deviation for the chromosomes in which they are located in sim-sec and higher divergences than the average plus standard deviation in sim-mel and sec-mel. Screening 2 selects genes that retained ancestral features and diverge significantly form *D. melanogaster*. Note that screening 2 appears more stringent than screening 1. Note also that a large number of common genes that can be found by both screenings in the autosomes, but considerably less on the X. No ancestral alleles could be found on the fourth chromosome (see text).

### Genes that diverge linearly and non-linearly in *D. simulans* and *D. sechellia*

In the second strategy to identify ancestral alleles, we searched for patterns of divergence among the 8,416 coding sequences. In this case, the data was sorted in ascending order of identity, and the values of the sim-sec pair were plotted against the sim-mel pair for the X chromosome, 2R and 2L, 3R and 3L chromosome arms (Fig. 2). Since the divergences of sim-mel and sec-mel are approximately the same (data not shown), the graphs of the sec-mel pair were not included in the figure.

The graphs in Figure 2 show that the genes that diverge the least in sim-sec pair, but also diverge the most in sim-mel pair fall between values 0 to 5% of the abscissa. Moreover, it is clear that the majority of genes also fall roughly within the ordinate range of 0 to 10%, and that within this interval there is a linear fit (Fig. 2, P<0.0001). We refer this interval to as *initial linear interval* (Fig. 2 A–E, outlined in red). Conversely, for values above 10% in the ordinate there is no significant agreement with a linear fit (P>0.05). We refer this interval to as *initial non-linear interval* (Fig. 2 A–E, outside the blue region). These results suggest that within the initial linear interval, the sim-sec genes diverge from sim-mel fairly linearly, while within the initial non-linear interval, this linearity breaks down. Thus, the non-linear interval contains the genes that varied the least in sim-sec and the most in sim-mel.

The results above suggest the existence of at least two gene populations; one large group that changes at a similar pace over generations in *D. simulans* and *D. sechellia* and a smaller group with a high degree of divergences.

### Delimiting a quadrant with genes that diverged from *D. melanogaster* and were inherited in *D. simulans* and *D. sechellia*

In the graphs shown in Fig. 2, we noticed the presence of a linear and a non-linear interval, but it is difficult to determine the boundary between the two intervals. In order to define more precisely this limit, we divided the data of sim-mel, sim-sec and sec-mel in percentiles, as shown in Fig. 2A′ for the X chromosome. Since each point in the graph represents one percentile, the line that transects the largest number of linear points along this curve reveals the percentage of genes that diverge linearly. On the X chromosome, this range corresponds to the 51^st^ percentile. Thus, the linearly distributed genes fall between the abscissa values 0 to 1.56 (sim-sec) and ordinate values 0 to 4.35 (sim-mel) (Fig. 2A′). We also applied the same methodology for the autosomes (data not shown).

We identified a more approximate interval where the genes diverge linearly in the abscissa and ordinate (Fig. 2, the inferior left quadrants in blue), and selected the genes that are in the quadrant above in the ordinate (Fig. 2, highlighted in gray). Using the same methodology for the pairs sim-sec and sec-mel, we identified the common set of genes in both searches (i.e. common genes to the percentiles of sim-sec vs. sim-mel and sim-sec vs. sec-mel).

A total of 439 genes common to *D. simulans* and *D. sechellia* were identified: 101 genes on the X, 74 on 2L, 89 on 2R, 77 on 3L and 98 on 3R (Table 1, Fig. 2). We were unable to perform a similar analysis for the fourth chromosome due to the fact that only 19 genes of high confidence were identified, which precluded the use of percentiles.

### Both screening strategies identify a large number of common genes

When the results of the percentile search are combined with the results of the search of averages and standard deviations, we notice that a significant number of genes are represented in both searches. In particular, 73 (98.6%) genes common to both searches were found on 2L; 76 (85.4%) on 2R, 61(79.2%) on 3L; and 96 (98.0%) on the 3R. Interestingly, on the X chromosome only 14 (13.9%) genes common to both searches were found (Table 1). The relatively low percentage of common genes observed on the X chromosome stems from the fact that the average divergence plus the standard deviation on this chromosome is higher than in the autosomes (Fig. 2, note the different spacing between the dashed line and solid lines on the X with autosomes). This variation results in the exclusion of several genes found by the method of percentiles and at the same time, in the inclusion of others. Thus, when the results of both screening are combined, the number of genes found by the averages method on the X chromosome is inferior to those found by the percentiles method. The main conclusions that can be drawn from these results are that both searches identify a large number of common genes and that the X chromosome genes evolve slightly faster than autosomal genes.

### Most genes identified are orthologous

To test whether the 320 genes identified in *D. simulans* and *D. sechellia* by both methods correspond to true orthologues as opposed to paralogues, we blasted the *D. simulans* and *D. sechellia* genes separately against the genome of *D. melanogaster*. Out of these 320 genes, 307 genes from each species matched the chromosomal position and gene used in *D. melanogaster* at the beginning of the screening. Thus, 96% of these genes correspond to homologues, not paralogs. The remaining 13 (4%) were excluded since they correspond to paralogs.

### Spatial distribution of ancestral alleles within the genome and divergence hotspots

**Figure 3:**
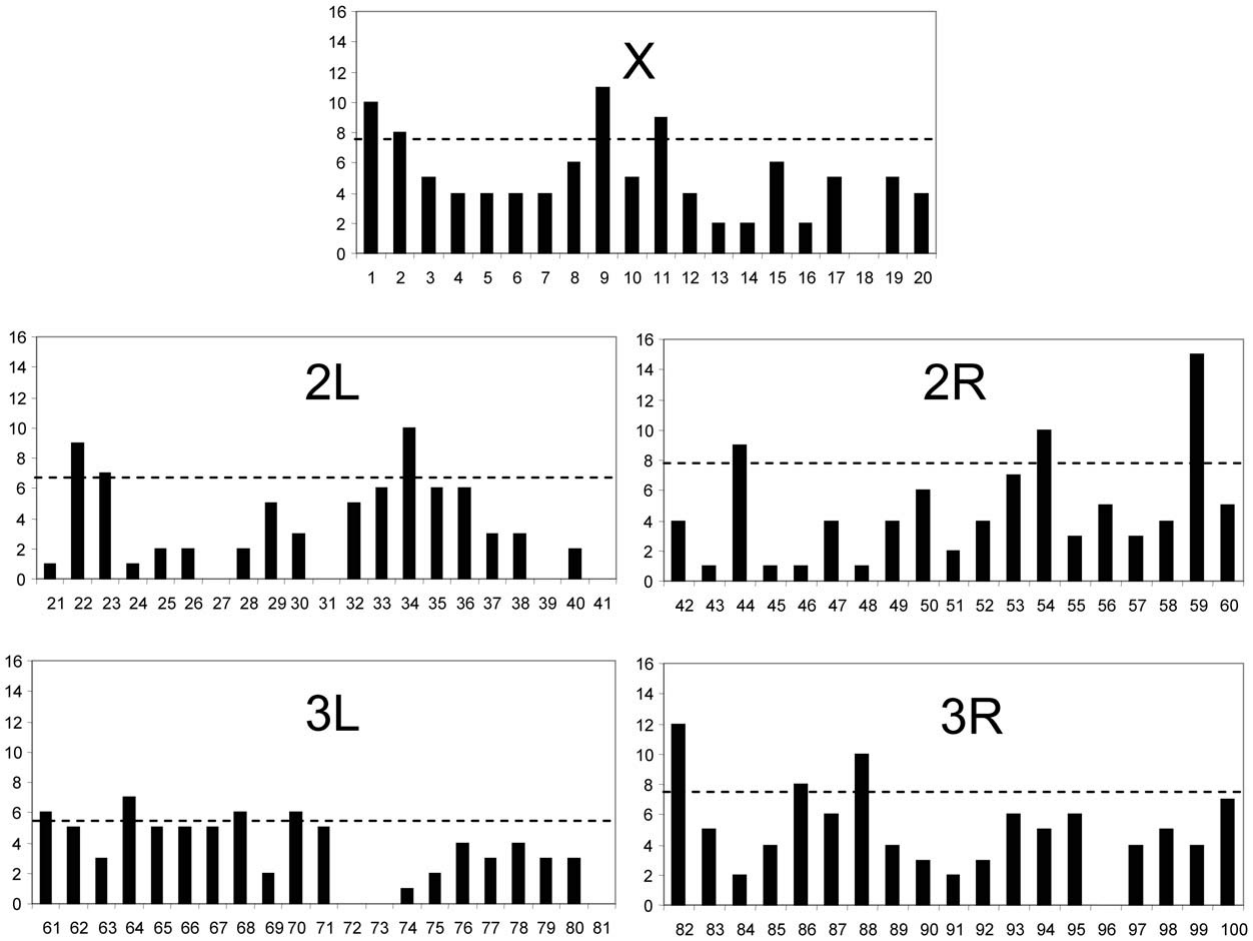
Distribution of ancestral alleles in the three major chromosomes. The 20 division coordinates used are those of *D. melanogaster*. Almost all divisions have one or more ancestral alleles. The dashed lines indicate the average number of alleles plus one standard deviation. Note that some divisions have a higher density of ancestral alleles than others.

We next tested whether the ancestral alleles are clustered in specific genomic locations or whether they appear evenly distributed across the genome. To address this issue we plotted their occurrence along the 20 divisions of the major chromosomes, using chromosomal coordinates of *D. melanogaster* [20] (Fig. 3). Since the method of averages and standard deviations produces a distortion that results in fewer common genes on the X chromosome, we used the results obtained from the percentile search to plot the position of these genes. Our data suggest that although these alleles can be found in almost every division of the three major chromosomes, some regions are hotspots for ancestral alleles. These regions were identified by searching for regions that have more ancestral alleles than the average plus 1 standard deviation. In particular, the X chromosome division 1 and division 9 have more ancestral alleles than most divisions on this chromosome. Similarly, three divisions on the left arm of the second chromosome (i.e. 22, 23 and 34) also harbor more ancestral alleles than the average plus one standard deviation for this arm. The right arm of the second chromosome also seems to have three hotspots in divisions 44, 54 and 59, while the distribution of ancestral alleles on the left arm of the third chromosome does not contain prominent hotspots, except perhaps by divisions 61, 64, 68 and 70. Finally, on the right arm of the third chromosome, one prominent hotspot appears at division 82. The significance of this clustering is not yet clear, but we note that some of these hotspots are located nearby known rearrangement breakpoints observed in *D. simulans* such as in divisions 1–2, divisions 21–22, 59–60 and 82 [21].

### Annotated biological functions of ancestral alleles

To test whether the genes identified have functions consistent with roles in species differentiation, we cross-referenced them to Gene Ontology (GO). If ancestral alleles participated in the segregation of these species, then we should expect to find biological functions consistent with pre and post-zygotic barriers, such as those that interfere with mating and cause interspecific lethality, sterility and mitotic defects in hybrids.

**Figure 4:**
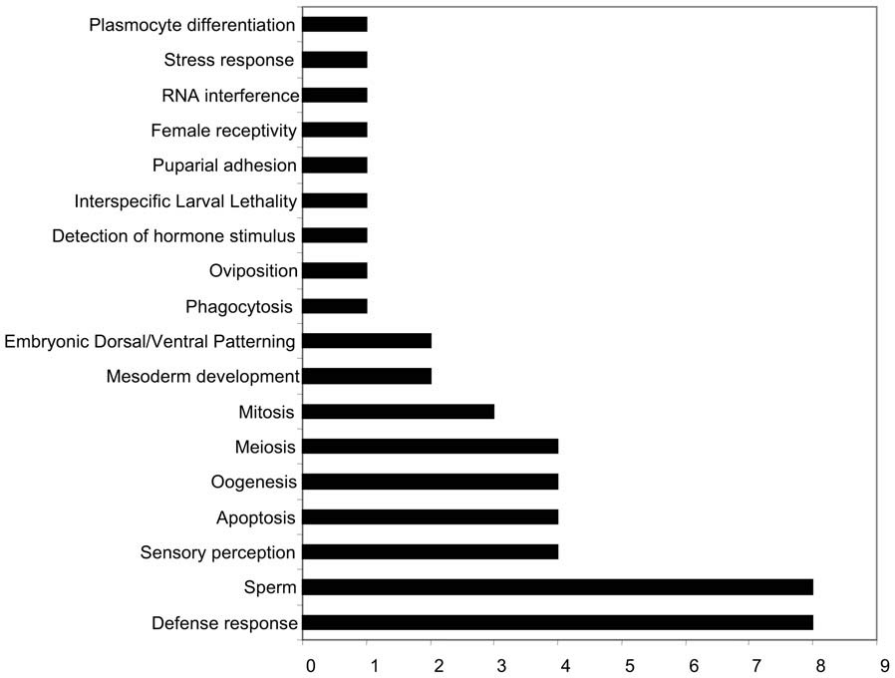
The biological functions of 48 ancestral alleles defined by Gene Ontology. The graph shows only genes with biological functions assigned by assays, inferred by sequence similarity or phenotype and does not include general biochemical properties such as phosphorylation, transcription initiation, signal transduction, proteolysis among others. The complete list of genes can be found in supporting information.

The GO referencing shows that despite the fact that less than 40% of these genes have either known molecular or biological functions (Table S1, Table S2, Table S3, Table S4 and Table S5), several ancestral alleles fall in discrete GO functional groups such as hybrid lethality, oogenesis, gamete generation, female meiosis, sperm competition and displacement, chemical perception of taste and olfaction, and immunity (Fig. 4, and in supporting information tables S1, S2, S3, S4 and S5).

Among known genes that cause zygotic barriers, our search readily identified *Lethal hybrid rescue* (*Lhr*) as an ancestral allele. The wild type allele of *Lhr* in *D. simulans* is responsible for the larval lethality in the sons of *D. simulans* males and *D. melanogaster* females [15], [19]. Similarly, the gene *CG14781* appears as an ancestral allele. *CG14781* has been implicated in mitotic spindle elongation and recently shown to correspond to *mei-38* [22], [23]. Null mutants of *mei-38* cause abnormal meiotic non-disjunction in females, abnormal mitosis and consequently lethality due to aneuploidy [23]. Thus, *mei-38* could be potentially involved in the sex-specific offspring hybrid lethality in females.

Our search also identified a number of genes with functions consistent with the formation of pre-zygotic barriers. For instance, accessory gland proteins such as Acp29AB and Acp98AB appear as ancestral alleles and it has been suggested that Acp29AB confers a resistance to the sperm of one male to be displaced by the sperm of another male, while Acp98AB appears to negatively regulate female receptivity [24], [25]. We also found genes involved in perception of taste such as *Gr59d* and *Gr59f* and odors like the *Odorant binding proteins Obp19a*, *Obp22a* and *Obp47a* [26]. These genes have been implicated in the sensory perception of chemical stimuli [27] and could potentially participate in the formation of pre-mating barriers through species-specific mate recognition.

Finally, our search identified ancestral alleles that potentially have novel and previously unsuspected functions such as immunity (Fig. 4). Together, these results suggest that the segregation of an ancestral population into three distinct species involved changes in reproduction, embryonic development, nervous system development and physiology, and immunity.

## Discussion

### Genomes and footprints of evolution

Extensive circumstantial evidences suggest that the genes that once created a sharp barrier between the ancestor of *D.melanogaster* and its sibling species might share an unusual conservation in *D. simulans* and *D. sechellia*. We tested this hypothesis by comparing their coding sequences and found 439 genes with little divergence in *D. simulans* and *D. sechellia*, but that diverge significantly from *D. melanogaster*.

The ancestral alleles identified in this work possibly record the earliest events in the differentiation of these *Drosophila* lineages that can be detected in extant species. The fact that these genes are very similar in *D. simulans/D. sechellia* but diverged from *D. melanogaster* more than most genes in the genome suggests two possible scenarios. In the first, the high divergence of ancestral alleles was acquired focally in time (i.e. this divergence is the result of one or few events that happened in short periods of time). The second possible scenario is that they were acquired over a longer period of time. (i.e. these genes are more prone to mutations and evolve faster than other genes). There are at least two evidences that favor the first hypothesis, but these are not yet conclusive. If these alleles were more prone to mutations, then we should expect that they would continue diverging at high rates after the separation of *D. simulans* from *D. sechellia*, but we did not observe such continuing divergence in the genome samples currently available. Also, if these alleles were more prone to mutations, then we should expect to observe high rates of polymorphism in *D. melanogaster* and *D. simulans*, which has not been reported in genomic results of different strains sequenced yet. In addition, we found a higher frequency of ancestral alleles near known chromosomal rearrangements, which raises the interesting possibility that these alleles could have been generated at the time those rearrangements appeared.

### The high divergence of X-chromosome genes, recombination and segregation patterns

In our search, we analyzed each chromosome separately to test whether there were variations in the rate of divergence among distinct chromosomes. The existence of such differences might provide an insight into the mechanism involved in the generation of ancestral alleles. Our analysis reveals that the average divergence plus the standard deviation for the X chromosome genes is higher than that of autosomal genes. Conversely, the same analysis suggests that the fourth chromosome has a lower average divergence than the other autosomes and the X chromosome. Together, these results show that the chromosome X evolves faster than the other autosomes and suggest that the fourth chromosome evolves slower. Since the rates of mutation and recombination on the X chromosome and in the two large autosomes do not appear significantly different [28] (http://flybase.org/maps/chromosomes/maps.html), the discrepancy between the divergences of the X and the remaining chromosomes is intriguing. However, this discrepancy possibly stems from the fact that the X chromosome is the only chromosome that exists in one or two copies (X/X and X/Y) in every generation. The existence of a hemizygote state allows recessive mutations on the X chromosome to be subject to the scrutiny of natural selection at least one generation before and in more individuals per generation, than a similar recessive mutation in an autosome. Thus, even with the same rate of mutation, recessive mutations on the X chromosome are subject to more rounds of selection than mutations in the autosomes, and consequently should have a better chance to become fixed.

### The difference in the mean plus standard deviation on the fourth chromosome genes suggest a possible mechanism for the generation of ancestral alleles

While analyzing the fourth chromosome, we detected an unusually low divergence in this chromosome. One possible explanation for this low divergence is that this is the only autosome that does not recombine during meiosis. Without recombination, errors acquired due to abnormal crossover are almost inexistent and the possibility of combining in a single chromosome different alleles floating in a population is equally low. Thus, the lack of errors during recombination and the combination of these mutations in a single chromosome could be accountable to some extent for the low levels of generation and accumulation of ancestral alleles on the fourth chromosome. However, since only 19 out of the 85 genes on this chromosome could be analyzed, this hypothesis needs to be more thoroughly tested as new high quality sequences become available for this chromosome.

### The advantages and limits of the analysis of ancestral alleles

The literature of speciation mechanisms has some examples of cleverly designed experiments to isolate genes required to block gene flow among closely related species. However, despite the fact that these screenings are of great significance and provide invaluable information about the approximate position of genes involved in speciation, researchers often face tremendous challenges to identify them molecularly. A typical example is *Lhr*, a gene identified genetically in 1979, which was only molecularly cloned almost 30 years later. This gene was readily identified as an ancestral allele in our search.

Our search can also potentially simplify the identification of other genes involved in speciation. For instance, Sawamura and cols. (2004) genetically mapped a female sterile mutant from *D. simulans*, presumably involved in the sterility observed in *D. melanogaster*/*D. simulans* hybrids, near the chromosomal division 32. Despite their efforts to narrow the region down to a 170 kb interval containing 20 coding sequences, they could not identify molecularly which of those 20 genes had a major effect on female fertility [29]. Our screening has identified 5 ancestral alleles on subdivision 32, and within the interval identified by Sawamura et al, there are only two ancestral alleles:Vm32E and CG14926. The GO of Vm32E suggests a role in the formation of embryonic vitelline membrane, which is consistent with female sterility, while CG14926 has no defined function but is expressed in male spermatocytes.

Although our analysis can potentially simplify the search and characterization of novel genes involved in speciation, there are some limits to its capabilities. The first one is the quality of the sequenced genomes. For instance, our search failed to identify *Hybrid male rescue*, since this gene does not have the same coverage in *D. simulans* and *D. sechellia* and for this reason was excluded from our analysis. Several other genes in the genome of *D. simulans* and *D. sechellia* also have a poor coverage. We expect that the search of evolutionary genes using the strategy outlined here will be greatly improved as more sequence gaps are filled.

The sequence comparison tools developed in this work can also be used in other types of screenings to identify genes involved in other biological processes unique to each sibling *Drosophila* species. For instance, since our screening was designed to identify ancestral alleles of *D. simulans* and *D. sechellia*, it eliminated genes required for particular specializations in each species. Our screening most likely missed genes that might be necessary for the feeding habits that make *D. simulans* and *D. melanogaster* cosmopolitan and *D. sechellia* restricted to *Morinda*. To identify the genes required for such differences, it would be necessary to screen for highly conserved genes in *D. simulans* and *D. melanogaster* that diverged in *D. sechellia*. Similarly, genes involved in the female choice for males would be expected to be missed by the current screening since females of the three species prefer males of their own species. To identify these genes the search should be directed to fast evolving genes (i.e. genes that are most divergent in the three species). Together, our results identify a relatively small number of genes that can be tested for speciation roles among *D. melanogaster* sibling species.

## Materials and Methods

### Gene extraction and searches

Usually genome searches that aim to find variation in coding sequences focus on translations since non-synonym amino acid variation is generally believed to produce phenotypic variation. However, this approach eliminates synonym substitutions that result in protein variation (e.g. mutations in splicing enhancers). For this reason, here we took all nucleotide variation in consideration.

Annotated sequences from the *Drosophila* library NT corresponding to the X (AE014298), 2L (AE014134), 2R (AE013599), 3L (AE014296), 3R (AE014297) and 4 (AE014135) arms or chromosomes were downloaded from the NCBI website and the coding sequences (CDS) were extracted using Extractor, a software developed by us. The extracted genes were then Blasted against the Whole Genome Sequences of *D. melanogaster* (mel-mel), *D. simulans* (mel-sim) and *D. sechellia* (mel-sec) obtained from the Whole Genome Shotgun (WGS) NCBI's library (14-Mar-2008). This library contains sequences from different strains of *D. simulans* and thus provides samples of gene variation in different populations. We developed another program, the Analyst, to automatically assemble the blast hits of the clone with best coverage using the *D. melanogaster* positions of the annotated coding sequences as a template. The Analyst also reported the coverage of all coding genes in *D. simulans* and *D. sechellia* and calculated the divergence between the pairs mel-sim, mel-sec and sim-sec by using their respective alignments.

### Blast settings and controls

Several different Blast settings were used in control experiments where annotated coding sequences of Drosophila were blasted against Whole Genome Sequences of *D. melanogaster*. These controls were used to define the Blast program that most consistently identifies the largest number of complete coding sequences in *D. melanogaster*. Discontinuous Megablast was chosen since it yielded the best reconstruction of the coding sequences in the control mel-mel.

### Triangulation of alleles with the same or similar nucleotide composition in *D. simulans* and *D. sechellia* that diverged from *D. melanogaster*

Identity values generated by Blast alignments provide information about the percentage of substitutions within a DNA segment, but not about the position of these substitutions. Thus, if a query gene in one species has the same identity of the subjects in two other species and this identity is different from 0, the two subjects may or may not contain mutations in the same position. To identify mutations in the same position, we triangulated the position of these substitutions by coverage and identity in pair wise comparisons between mel-sim, mel-sec and sim-sec. Using this system, genes that diverged significantly from *D. melanogaster* and were inherited by *D. simulans* and *D. sechellia* lineages should appear with the same coverage and an identical or similar identity in the *D. simulans*/*D. sechellia* comparison, and with equally fewer identities in the *D. melanogaster/D. simulans* and *D. melanogater/D. sechellia* comparisons.

To minimize errors due to the incorrect automatic assembly of the coding sequences in *D. simulans* and *D. sechellia* that could interfere with the evaluation of the divergence (i.e. truncated coding sequences due to the inability of Blast to identify particular exons), the coding sequences of *D. melanogaster* were Blasted against the *D. melanogaster* WGS library and the predicted coding sequences assembled from the Blast hits. The identities of these predicted coding sequences were then compared to the actual coding sequences in the annotated genome and only genes with at least 99% of identity with the annotated coding sequences were included in the analysis. To avoid false-positives due to incomplete clone representation, the data was sorted by coverage in sim and sec, and only genes with the same coverage were selected.

### Cross-referencing to gene function

The functional cross-referencing of the genes identified was done using annotated biological functions from flybase (http://flybase.bio.indiana.edu/), as well as descriptions in the literature.

## Supporting Information

Table S1 Gene Ontology terms of genes identified in the screening located on the X-chromosome.
(0.08 MB PDF)

Table S2 Gene Ontology terms of genes identified in the screening located on 2L.
(0.08 MB PDF)

Table S3 Gene Ontology terms of genes identified in the screening located on 2R.
(0.08 MB PDF)

Table S4 Gene Ontology terms of genes identified in the screening located on 3L.
(0.08 MB PDF)

Table S5 Gene Ontology terms of genes identified in the screening located on 3R.
(0.08 MB PDF)
