# Supplementary material for: An Analysis of Genetic Changes during the Divergence of Drosophila Species
Source: PLoS One. 2010 May 5;5(5):e10485. doi: 10.1371/journal.pone.0010485 (PMC2864749; doi:10.1371/journal.pone.0010485)
Supplement: Table S1 — Gene Ontology terms of genes identified in the screening located on the X-chromosome. (0.08 MB PDF) [file pone.0010485.s001.pdf]

GENE	GO BIOLOGICAL PROCESS
CG32816	biological_process | no biological data available
CG13369	D-ribose metabolic process | inferred from electronic annotation with InterPro:IPR002139
CG13362	-
CG14635	-
CG14633	-
TfIIA-S-2	transcription initiation from RNA polymerase II promoter | inferred from sequence or structural similarity with HGNC_gene:GTF2A2; HGNC:4647; OMIM:600519 AND inferred from sequence or structural similarity with SGD_LOCUS:TOA2; SGD:S0001541<br>regulation of transcription from RNA polymerase II promoter | non-traceable author statement
CG14631	-
CG33082	-
CG11381	-
CG14781	mitotic spindle elongation | inferred from mutant phenotype
CG32810	ion transport | inferred from sequence or structural similarity with NCBI_NP:508239<br>potassium ion transport | inferred from electronic annotation with InterPro:IPR003131
CG32803	-
CG3857	-
CG4325	-
CG14054	-
CG4061	RNA processing | inferred from electronic annotation with InterPro:IPR013791, InterPro:IPR017770
CG3091	transport | inferred from electronic annotation with InterPro:IPR001071
CG2662	-
CG10793	response to oxidative stress | inferred from electronic annotation with InterPro:IPR002016
CG14269	-
CG16782	-
Mnt	regulation of transcription | inferred from sequence or structural similarity with dm<br>phagocytosis, engulfment | inferred from mutant phenotype
CG15571	-
CG15576	-
CG15577	-
CG15786	-
CG15768	proteolysis | inferred from electronic annotation with InterPro:IPR000209, InterPro:IPR000834
CG15764	-
CG12236	-
CG6041	proteolysis | inferred from electronic annotation with InterPro:IPR001254, InterPro:IPR001314, InterPro:IPR018114
CG17717	-
CG3226	-
CG4575	regulation of transcription, DNA-dependent | inferred from electronic annotation with InterPro:IPR004827, InterPro:IPR011700
inx7	-
CG1999	-
CG15330	-
CG2256	-
CG2147	-
CG12661	-
CG10970	-
Dip1	-
CG12057	-
CG34339	-
CG15317	-
Gga	post-Golgi vesicle-mediated transport | inferred from sequence or structural similarity with UniProtKB:Q9UJY5, UniProt:Q9UJY4, UniProt:Q9NZ52<br>intracellular protein transport | inferred from electronic annotation with InterPro:IPR002014, InterPro:IPR004152, InterPro:IPR008152, InterPro:IPR013041
CG1791	signal transduction | inferred from electronic annotation with InterPro:IPR002181, InterPro:IPR014716
Gip	-
l(1)G0289	-
CG2898	-
RabX2	regulation of transcription, DNA-dependent | inferred from electronic annotation with InterPro:IPR002078<br>small GTPase mediated signal transduction | inferred from electronic annotation with InterPro:IPR001806, InterPro:IPR003579<br>protein transport | inferred from electronic annotation with InterPro:IPR003579
CG15296	-
CG2124	-
CG12637	-
CG15209	-
CG17333	pentose-phosphate shunt | non-traceable author statement
CG11106	-
CG11756	-
CG1738	-
CG9360	metabolic process | inferred from electronic annotation with InterPro:IPR002198, InterPro:IPR002347, InterPro:IPR016040
CG15739	metabolic process | inferred from electronic annotation with InterPro:IPR005834, InterPro:IPR006357
CG11356	-
CG15927	-
CG1463	-
CG2543	tetrahydrofolylpolyglutamate biosynthetic process | inferred from sequence or structural similarity with UniProtKB:Q05932
mRpL49	translational initiation | inferred from electronic annotation with InterPro:IPR001950
CG32643	-
CG4351	-
CG15747	-
CG12725	protein modification process | inferred from electronic annotation with InterPro:IPR000626
CG12481	-
CG13403	-
CG12540	-
nmdyn-D6	CTP biosynthetic process | inferred from electronic annotation with InterPro:IPR001564<br>UTP biosynthetic process | inferred from electronic annotation with InterPro:IPR001564<br>GTP biosynthetic process | inferred from electronic annotation with InterPro:IPR001564
CG15641	transport | inferred from electronic annotation with InterPro:IPR001588
CG15601	-
CG12395	-
CG34015	biological_process | no biological data available
CG4653	proteolysis | inferred from electronic annotation with InterPro:IPR001254, InterPro:IPR001314, InterPro:IPR018114
CG4789	regulation of transcription, DNA-dependent | inferred from electronic annotation with InterPro:IPR002078
CG13004	-
CG4955	-
CG18258	lipid metabolic process | inferred from electronic annotation with InterPro:IPR000734
CG8915	-
CG8408	biological_process | no biological data available
CG12672	-
Her	negative regulation of transcription from RNA polymerase II promoter | inferred from sequence or structural similarity with MGD:Hes1; MGI:MGI:104853
CG15043	-
CG15047	-
CG6873	-
CG34329	-
CG1504	-
Obp19a	olfactory behavior | inferred from sequence or structural similarity with UniProtKB:O02372<br>response to pheromone | inferred from sequence or structural similarity with UniProtKB:O02372<br>sensory perception of chemical stimulus | inferred from sequence or structural similarity with UniProtKB:P34174<br>transport | inferred from electronic annotation with InterPro:IPR006170
CG1324	-
CG1304	proteolysis | non-traceable author statement
CG1722	-
CG41106	biological_process | no biological data available
FucTC	protein amino acid glycosylation | inferred from electronic annotation with InterPro:IPR001503
Rab21	small GTPase mediated signal transduction | inferred from electronic annotation with InterPro:IPR001806, InterPro:IPR003579<br>protein transport | inferred from electronic annotation with InterPro:IPR003579
CG13865	biological_process | no biological data available
