# Supplementary material for: An Analysis of Genetic Changes during the Divergence of Drosophila Species
Source: PLoS One. 2010 May 5;5(5):e10485. doi: 10.1371/journal.pone.0010485 (PMC2864749; doi:10.1371/journal.pone.0010485)
Supplement: Table S2 — Gene Ontology terms of genes identified in the screening located on 2L. (0.08 MB PDF) [file pone.0010485.s002.pdf]

GENE	GO BIOLOGICAL PROCESS
Iris	oogenesis | non-traceable author statement
CG17650	-
CG7295	-
CG33673	biological_process | no biological data available
Obp22a	sensory perception of chemical stimulus | inferred from sequence or structural similarity with UniProtKB:P34174<br>transport | inferred from electronic annotation with InterPro:IPR006170
CG12674	-
CG4259	proteolysis | inferred from electronic annotation with InterPro:IPR001254
CG17239	proteolysis | non-traceable author statement
CG31949	biological_process | no biological data available
CG3515	biological_process | no biological data available
CG33125	biological_process | no biological data available
CG17301	ubiquitin-dependent protein catabolic process | inferred from sequence or structural similarity with UniProtKB:P40307
CG15398	regulation of transcription, DNA-dependent | inferred from electronic annotation with InterPro:IPR000814<br>transcription initiation from RNA polymerase II promoter | inferred from electronic annotation with InterPro:IPR000814
CG9641	-
CG17261	-
CG8838	-
CG31776	oligosaccharide biosynthetic process | inferred from direct assay
lectin-24A	-
Umbrea - unknown ID	
Jon25Bii	proteolysis | non-traceable author statement
CG13989	-
CG31633	-
r2d2	RNA interference, siRNA loading onto RISC | inferred from direct assay<br>defense response to virus | inferred from mutant phenotype
CG33121	-
Acp29AB	sperm displacement | non-traceable author statement<br>sperm competition | traceable author statement<br>post-mating behavior | non-traceable author statement
lectin-29Ca	-
CG31898	biological_process | no biological data available
CG13088	-
CG9573	-
CG4438	-
CG31882	biological_process | no biological data available
CG18854	-
CG34043	biological_process | no biological data available
CG7300	-
CG6444	-
Vm32E	vitelline membrane formation in chorion-containing eggshell | non-traceable author statement
CG14926	-
CG6555	-
CG14944	cyclic nucleotide metabolic process | inferred from sequence or structural similarity with UniProtKB:Q63421<br>regulation of proteolysis | inferred from sequence or structural similarity with UniProtKB:P34953<br>signal transduction | inferred from electronic annotation with InterPro:IPR002073
CG12314	-
CG14947	-
Pkd2	sperm competition | inferred from mutant phenotype<br>sperm motility | inferred from mutant phenotype<br>larval feeding behavior | inferred from mutant phenotype

	smooth muscle contraction | inferred from mutant phenotype
CG31858	biological_process | no biological data available
ref2	mRNA export from nucleus | non-traceable author statement
Pk34A	protein amino acid phosphorylation | inferred from sequence or structural similarity
CG16957	-
CG16956	-
CG16852	-
CG31845	biological_process | no biological data available
CG16888	-
He	innate immune response | inferred from mutant phenotype<br>encapsulation of foreign target | traceable author statement<br>negative regulation of lamellocyte differentiation | traceable author statement
CG33681	biological_process | no biological data available
CG15293	-
CG31769	biological_process | no biological data available
CG15286	-
CG18125	proteolysis | inferred from electronic annotation with InterPro:IPR001254, InterPro:IPR001314, InterPro:IPR018114
CG15262	regulation of transcription | inferred from electronic annotation with InterPro:IPR007282
CG18063	-
CG17329	-
CG31737	-
CG4631	-
squ	dorsal appendage formation | inferred from mutant phenotype<br>oogenesis | inferred from mutant phenotype
CG33552	biological_process | no biological data available
CG12620	regulation of phosphoprotein phosphatase activity | inferred from electronic annotation with InterPro:IPR007062<br>regulation of signal transduction | inferred from electronic annotation with InterPro:IPR007062
CG31804	biological_process | no biological data available
CG15172	-
CG17344	-
CG17549	-
CG34051	biological_process | no biological data available
CG16772	-
CG15475	-
CG31601	biological_process | no biological data available
CG5922	-
