# Supplementary material for: An Analysis of Genetic Changes during the Divergence of Drosophila Species
Source: PLoS One. 2010 May 5;5(5):e10485. doi: 10.1371/journal.pone.0010485 (PMC2864749; doi:10.1371/journal.pone.0010485)
Supplement: Table S3 — Gene Ontology terms of genes identified in the screening located on 2R. (0.08 MB PDF) [file pone.0010485.s003.pdf]

| GENE | GO BIOLOGICAL PROCESS |
|---|---|
| CG30432 | biological_process \| no biological data available |
| CG15234 | - |
| CG9445 | mesoderm development \| inferred from expression pattern |
| Tsp42Ee | - |
| CG11112 | - |
| CG8712 | - |
| coilin | - |
| CG30374 | proteolysis \| inferred from electronic annotation with InterPro:IPR001254 |
| CG14756 | - |
| CG14759 | - |
| CG34219 | - |
| CG8237 | - |
| CG8229 | - |
| CG13742 | - |
| CG30008 | - |
| CG30017 | biological_process \| no biological data available |
| Obp47a | sensory perception of chemical stimulus \| inferred from sequence or structural similarity with UniProtKB:P34174<br>transport \| inferred from electronic annotation with InterPro:IPR006170 |
| CG34224 | - |
| Cpr47Eg | transport \| inferred from electronic annotation with InterPro:IPR001588 |
| CG13190 | biological_process \| no biological data available |
| CG13159 | - |
| CG33792 | biological_process \| no biological data available |
| CG4616 | - |
| CG4716 | - |
| CG17048 | - |
| CG17050 | - |
| CG17049 | - |
| CG34236 | - |
| CG8589 | - |
| CG10102 | - |
| CG12857 | - |
| Cyp6a21 | - |
| CG30472 | biological_process \| no biological data available |
| CG30466 | biological_process \| no biological data available |
| CG30090 | proteolysis \| inferred from electronic annotation with InterPro:IPR001254, InterPro:IPR001314, InterPro:IPR018114 |
| CG33460 | - |
| CG30099 | - |
| CG15710 | - |
| CG15711 | - |
| CG33459 | proteolysis \| inferred from electronic annotation with InterPro:IPR001254, InterPro:IPR001314, InterPro:IPR018114 |
| CG4927 | proteolysis \| non-traceable author statement |
| Acp53C14c | - |
| tef | male meiosis I \| inferred from mutant phenotype<br>synapsis \| non-traceable author statement<br>male meiosis chromosome segregation \| inferred from mutant phenotype |
| CG11400 | - |
| CG15917 | - |
| CG10950 | protein import into nucleus \| inferred from sequence or structural similarity with SGD_LOCUS:MTR10; SGD:S0005686 |
| Lhr | - |
| CG34192 | - |
| CG6520 | - |
| Dcr-2 | RNA interference \| inferred from direct assay<br>RNA interference, production of siRNA \| inferred from direct assay<br>RNA interference, siRNA loading onto RISC \| inferred from mutant phenotype<br>RNA interference, targeting of mRNA for destruction \| inferred from mutant phenotype<br>chromatin silencing \| inferred from mutant phenotype<br>defense response to virus \| inferred from mutant phenotype |

| | |
|---|---|
| CG18186 | chromatin assembly or disassembly \| inferred from electronic annotation with InterPro:IPR000953 |
| CG10934 | - |
| CG10931 | - |
| PpY-55A | protein amino acid dephosphorylation \| inferred from sequence or structural similarity |
| CG14502 | - |
| l(2)08717 | wing disc dorsal/ventral pattern formation \| inferred from genetic interaction with Bx, ap |
| CG18605 | - |
| CG7137 | - |
| CG15124 | - |
| CkIIbeta2 | protein amino acid phosphorylation \| inferred from sequence or structural similarity |
| CG13871 | - |
| CG15657 | - |
| CG30391 | - |
| CG30283 | proteolysis \| inferred from electronic annotation with InterPro:IPR001254, InterPro:IPR001314, InterPro:IPR018114 |
| comr | positive regulation of transcription from RNA polymerase II promoter \| inferred from mutant phenotype<br>spermatogenesis \| inferred from mutant phenotype |
| CG11362 | - |
| CG4294 | phosphate metabolic process \| inferred from electronic annotation with InterPro:IPR008162 |
| CG13512 | biological_process \| no biological data available |
| CG30270 | - |
| CG30187 | proteolysis \| inferred from electronic annotation with InterPro:IPR001254, InterPro:IPR001314, InterPro:IPR018114 |
| CG32833 | proteolysis \| inferred from electronic annotation with InterPro:IPR001254 |
| Gr59d | sensory perception of taste \| inferred from sequence or structural similarity |
| Klp59C | mitotic sister chromatid segregation \| inferred from mutant phenotype<br>microtubule-based movement \| inferred from sequence or structural similarity with EMBL:Y15894; protein_id:CAA75815 |
| CG3162 | nuclear mRNA splicing, via spliceosome \| inferred from sequence or structural similarity with UniProtKB:P26369 |
| RpL22-like | translation \| inferred from sequence or structural similarity with HGNC_gene:RPL22; HGNC:10315; OMIM:180474 |
| CG12782 | poly(A)+ mRNA export from nucleus \| inferred from sequence or structural similarity with UniProtKB:P41838 |
| CG13541 | - |
| CG9863 | - |
| CG30409 | - |
| CG13557 | - |
| CG13559 | - |
| Gr59f | sensory perception of taste \| inferred from sequence or structural similarity |
| IM18 | defense response \| inferred from direct assay |
| CG15800 | ubiquitin-dependent protein catabolic process \| inferred from electronic annotation with InterPro:IPR001232, InterPro:IPR016072 |
| CG30177 | biological_process \| no biological data available |
| CG13589 | - |
| CG13590 | - |
| CG33680 | detection of hormone stimulus \| inferred from sequence or structural similarity with InterPro:IPR013053 |
