# Supplementary material for: An Analysis of Genetic Changes during the Divergence of Drosophila Species
Source: PLoS One. 2010 May 5;5(5):e10485. doi: 10.1371/journal.pone.0010485 (PMC2864749; doi:10.1371/journal.pone.0010485)
Supplement: Table S4 — Gene Ontology terms of genes identified in the screening located on 3L. (0.08 MB PDF) [file pone.0010485.s004.pdf]

GENE	GO BIOLOGICAL PROCESS
CG13875	-
CG16971	biological_process | no biological data available
CG32845	-
CG1231	-
CG13884	-
CG13896	-
CG2199	-
CG32319	protein amino acid acetylation | inferred from sequence or structural similarity with UniProtKB:Q03503
CG13931	-
CG12035	-
CG11815	-
CG32308	biological_process | no biological data available
CG10862	regulation of protein metabolic process | inferred from electronic annotation with InterPro:IPR000608<br>post-translational protein modification | inferred from electronic annotation with InterPro:IPR000608
CG10855	-
mge	intracellular protein transport | inferred from electronic annotation with InterPro:IPR005683
CG10853	-
CG14983	-
CG32243	biological_process | no biological data available
CG32246	transport | inferred from electronic annotation with InterPro:IPR006061
CG13717	-
CG13716	regulation of localization | inferred from sequence or structural similarity with halo
CG32232	microtubule cytoskeleton organization | inferred from electronic annotation with InterPro:IPR007259
CG10478	protein import into nucleus | inferred from sequence or structural similarity with HGNC_gene:KPNA4; HGNC:6397; OMIM:6029702
l(3)mbn	plasmatocyte differentiation | non-traceable author statement
Acp65Aa	-
CG13297	-
CG7386	-
CG14835	-
sphinx2	innate immune response | inferred from mutant phenotype<br>defense response to Gram-positive bacterium | inferred from mutant phenotype<br>defense response to Gram-negative bacterium | inferred from mutant phenotype<br>defense response to fungus | inferred from mutant phenotype<br>positive regulation of Toll signaling pathway | inferred from mutant phenotype<br>proteolysis | inferred from electronic annotation with InterPro:IPR001254
CG7526	-
CG32371	-
CG7213	-
PGRP-LF	defense response | non-traceable author statement<br>innate immune response | non-traceable author statement<br>peptidoglycan catabolic process | non-traceable author statement
CG16719	mesoderm development | non-traceable author statement
CG14164	-
Nc	head involution | inferred from mutant phenotype<br>apoptosis | inferred from mutant phenotype<br>hemocyte development | inferred from mutant phenotype<br>compound eye development | inferred from mutant phenotype<br>central nervous system development | inferred from mutant phenotype<br>autophagic cell death | inferred from expression pattern<br>salivary gland cell autophagic cell death | inferred from expression pattern<br>apoptotic program | inferred from expression pattern AND inferred from mutant phenotype<br>induction of programmed cell death by hormones | traceable author statement<br>embryonic development via the syncytial blastoderm | non-traceable author statement<br>regulation of retinal cell programmed cell death | non-traceable author statement<br>sperm individualization | inferred from mutant phenotype<br>sensory organ development | inferred from mutant phenotype<br>metamorphosis | traceable author statement<br>determination of adult life span | inferred from mutant phenotype<br>protein autoprocessing | inferred from direct assay<br>zymogen activation | inferred from direct assay inferred from mutant phenotype<br>caspase activation | inferred from mutant phenotype<br>proteolysis | inferred from electronic annotation with InterPro:IPR001309, InterPro:IPR002138, InterPro:IPR002398, InterPro:IPR011600, InterPro:IPR016129
CG6527	-
CG6185	-
CG6168	proteolysis | inferred from sequence or structural similarity<br>defense response to Gram-negative bacterium | inferred from mutant phenotype
CG11714	-
CG11570	-

CG17154	-
CG17666	-
CG32110	proteolysis | inferred from sequence or structural similarity with UniProtKB:Q9P0U3
CG32135	mRNA export from nucleus | inferred from sequence or structural similarity with UniProtKB:O88984
CG17359	-
CG32141	biological_process | no biological data available
CG13482	-
CG3919	-
CG13476	-
CG18581	-
CG13463	-
CG13460	-
ran-like	intracellular protein transport | inferred from electronic annotation with InterPro:IPR002041<br>signal transduction | inferred from electronic annotation with InterPro:IPR002041<br>nucleocytoplasmic transport | inferred from electronic annotation with InterPro:IPR002041
Eig71Ed	autophagic cell death | inferred from expression pattern<br>salivary gland cell autophagic cell death | inferred from expression pattern
CG33690	biological_process | no biological data available
CG13732	-
CG4306	-
CG34254	-
CG14089	-
825-Oak	biological_process | no biological data available
CG33710	biological_process | no biological data available
schuy	-
CG7298	chitin metabolic process | inferred from electronic annotation with InterPro:IPR002557
CG6951	-
CG11451	mitotic spindle organization | inferred from mutant phenotype
CG12984	-
CG7370	-
CG7145	proline metabolic process | inferred from sequence or structural similarity with UniProtKB:P30038<br>proline biosynthetic process | inferred from electronic annotation with InterPro:IPR005931
CG15374	-
CG11404	-
CG14453	-
CG14451	-
CG32461	biological_process | no biological data available
CG40294	-
