# Supplementary material for: An Analysis of Genetic Changes during the Divergence of Drosophila Species
Source: PLoS One. 2010 May 5;5(5):e10485. doi: 10.1371/journal.pone.0010485 (PMC2864749; doi:10.1371/journal.pone.0010485)
Supplement: Table S5 — Gene Ontology terms of genes identified in the screening located on 3R. (0.08 MB PDF) [file pone.0010485.s005.pdf]

GENE	GO BIOLOGICAL PROCESS
CG31516	biological_process | no biological data available
CG14639	-
CG41461	-
CG41468	-
CG31525	biological_process | no biological data available
CG31526	biological_process | no biological data available
CG41516	-
CG14659	-
CG14662	-
CG12590	-
CG12161	ubiquitin-dependent protein catabolic process | inferred from electronic annotation with InterPro:IPR000243, InterPro:IPR001353
mia	transcription initiation from RNA polymerase II promoter | inferred from sequence or structural similarity with HGNC_gene:TAF6L; HGNC:17305; OMIM:602946 AND inferred from sequence or structural similarity with SGD_LOCUS:TAF60; SGD:S0003080<br>male meiosis I | inferred from mutant phenotype<br>spermatogenesis | inferred from mutant phenotype<br>meiosis | traceable author statement<br>spermatid development | traceable author statement<br>regulation of transcription factor activity | inferred from electronic annotation with InterPro:IPR011442
CG31554	biological_process | no biological data available
CG1075	proteolysis | inferred from electronic annotation with InterPro:IPR000668
CG15185	-
CG1138	-
nxf4	RNA export from nucleus | inferred from sequence or structural similarity with NCBI_NP:071336
Mst84Dd	-
CG11977	-
CG33630	biological_process | no biological data available
CG34108	biological_process | no biological data available
CG14694	transport | inferred from electronic annotation with InterPro:IPR002666
CG14708	-
l-t	negative regulation of protein amino acid dephosphorylation | inferred from direct assay
CG10013	-
CG10096	metabolic process | inferred from electronic annotation with InterPro:IPR016040
CG17207	biological_process | no biological data available
CG33929	biological_process | no biological data available
CG10909	rRNA processing | inferred from sequence or structural similarity with SGD_LOCUS:NOP1; SGD:S0002172
CG14384	-
CG14370	-
CG14369	-
CG9925	-
CG14357	-
CG9616	-
CG14851	-
FK506-bp1	protein folding | inferred from sequence or structural similarity with UniProtKB:Q26486
CG5399	-
CG34274	-
CG12784	-
CG5614	-
ird5	positive regulation of antibacterial peptide biosynthetic process | inferred from mutant phenotype<br>protein catabolic process | inferred from direct assay<br>protein amino acid phosphorylation | non-traceable author statement<br>Toll signaling pathway | traceable author statement<br>dorsal/ventral axis specification | non-traceable author statement<br>antibacterial humoral response | inferred from mutant phenotype<br>antimicrobial humoral response | non-traceable author statement
CG31287	protein folding | inferred from electronic annotation with InterPro:IPR004127
Mst89B	-
CG17930	transport | inferred from electronic annotation with InterPro:IPR005828
CG10317	-
Actn3	actin filament bundle formation | inferred from sequence or structural similarity with EMBL:AF093775
CG5866	-
Sgs5	puparial adhesion | traceable author statement
CG34281	-
CG14315	-
CG14313	-
CG7669	-
CG14306	-
CG4538	ATP-dependent proteolysis | inferred from sequence or structural similarity with UniProtKB:O76031<br>protein transport | inferred from electronic annotation with InterPro:IPR004487
Pros28.1A	ubiquitin-dependent protein catabolic process | inferred from electronic annotation with InterPro:IPR000426, InterPro:IPR001353
TotC	response to stress | inferred from sequence or structural similarity with TotA
CheB93b	-
CG6690	oxidation reduction | inferred from electronic annotation with InterPro:IPR006863<br>cell redox homeostasis | inferred from electronic annotation with InterPro:IPR013766, InterPro:IPR017936
BG4	defense response | non-traceable author statement<br>signal transduction | non-traceable author statement<br>immune response | inferred from mutant phenotype<br>apoptosis | non-traceable author statement<br>sperm individualization | inferred from mutant phenotype<br>defense response to Gram-negative bacterium | traceable author statement<br>positive regulation of antibacterial peptide biosynthetic process | traceable author statement
CG17625	-
CG31281	-
p53	DNA damage response, signal transduction by p53 class mediator | traceable author statement<br>DNA damage response, signal transduction resulting in induction of apoptosis | traceable author statement<br>apoptosis | traceable author statement<br>cell death | inferred from mutant phenotype<br>response to radiation | inferred from mutant phenotype<br>DNA damage response, signal transduction by p53 class mediator resulting in induction of apoptosis | inferred from mutant phenotype<br>response to DNA damage stimulus | inferred from mutant phenotype<br>regulation of transcription from RNA polymerase II promoter | traceable author statement<br>determination of adult life span | inferred from mutant phenotype<br>tissue regeneration | inferred from mutant phenotype<br>response to UV | inferred from mutant phenotype<br>caspase activation | inferred from mutant phenotype<br>response to ionizing radiation | inferred from mutant phenotype<br>positive regulation of gene-specific transcription | inferred from mutant phenotype<br>positive regulation of cell cycle | inferred from genetic interaction with CoVa
CG31457	-
CG31515	-
CG10177	protein amino acid phosphorylation | non-traceable author statement<br>intracellular signaling cascade | inferred from electronic annotation with InterPro:IPR003533
CG10164	-
CG16710	proteolysis | non-traceable author statement
CG18754	proteolysis | non-traceable author statement
CG10254	regulation of protein metabolic process | inferred from electronic annotation with InterPro:IPR000608<br>post-translational protein modification | inferred from electronic annotation with InterPro:IPR000608
cav	telomere capping | inferred from expression pattern<br>telomere maintenance | inferred from mutant phenotype
CG31128	biological_process | no biological data available
bam	gamete generation | inferred from mutant phenotype

	cystoblast division | inferred from mutant phenotype<br>fusome organization | inferred from mutant phenotype<br>spermatogenesis | traceable author statement<br>germ-line stem cell division | inferred from mutant phenotype<br>germarium-derived female germ-line cyst formation | inferred from mutant phenotype<br>cell fate specification | traceable author statement<br>germ cell development | traceable author statement<br>male meiosis | traceable author statement<br>female germ-line cyst formation | traceable author statement<br>cell fate determination | traceable author statement<br>spectrosome organization | traceable author statement<br>spermatogonial cell division | traceable author statement<br>vesicle targeting to fusome | traceable author statement<br>oogenesis | inferred from mutant phenotype
CG11889	-
BG642167	-
Mst57Db	oviposition | non-traceable author statement<br>post-mating behavior | non-traceable author statement
CG14545	-
CG34130	biological_process | no biological data available
CG14244	-
CG14262	-
CG5402	-
CG12879	-
Acp98AB	negative regulation of female receptivity, post-mating | non-traceable author statement
CG33213	biological_process | no biological data available
CG5527	proteolysis | inferred from electronic annotation with InterPro:IPR000718, InterPro:IPR008753, InterPro:IPR018497
CG34436	-
CG15510	-
Jon99Fi	proteolysis | inferred from electronic annotation with InterPro:IPR001254, InterPro:IPR001314, InterPro:IPR018114
CG2267	-
CG31013	protein metabolic process | inferred from electronic annotation with InterPro:IPR006620<br>oxidation reduction | inferred from electronic annotation with InterPro:IPR013547
CG1340	translational initiation | inferred from sequence or structural similarity
CG31008	-
Prosalpha3T	ubiquitin-dependent protein catabolic process | inferred from electronic annotation with InterPro:IPR000426, InterPro:IPR001353
CG18672	one-carbon compound metabolic process | inferred from electronic annotation with InterPro:IPR018338
CG1792	-
